# FinTech adoption, HR competency potential, service innovation and firm growth in banking sector

**DOI:** 10.1016/j.heliyon.2023.e13967

**Published:** 2023-02-23

**Authors:** Sana Arz Bhutto, Yasir Jamal, Saif Ullah

**Affiliations:** a Department of Business Administration, ILMA University Karachi, Pakistan; b Business Administration Department, Mohammed Ali Jinnah University, Pakistan; c Department of Management, Technology and Information Sciences at Ziauddin University Karachi, Pakistan

**Keywords:** Human resource competencies, FinTech adoption, Service innovation, Firm growth, Banks

## Abstract

This study investigates the role of service innovation as a mediator between FinTech adoption and firm growth. Furthermore, the study also explores the role of human resource competency in FinTech adoption. Survey questionnaires were given to participants in this study, which used a quantitative methodology and were distributed to fifty-five United States banks. A sample of 311 responses was collected and analyzed using Structural Equation Modeling (SEM). The results show that human resource competencies such as creating, adapting (to change), deciding to initiate action and interpreting analysis positively impact FinTech adoption. This study also discovered that service innovation contributes to firm growth. The findings confirmed the influence of human resource competencies on FinTech adoption in banks. This study suggests implementing effective human resource practices to develop employee competencies. Employee performance can be optimized to impact service innovation and business growth, which promotes the adoption of FinTech. The research adds to the body of knowledge already available by providing evidence of mediating role between FinTech adoption and firm growth.

## 1 Introduction

Over the past few years, the financial services industry has focused on adopting FinTech as a strategic approach. That is why the financial services sector aims to identify the most appropriate competency potentials of the human resource, which can lead to the adoption of FinTech [1]. In this realm, competency potential has emerged as a fundamental concept that specifies a composition of competencies that includes the abilities, skills, and personalities considered to enhance employee performance [2]. It is worth mentioning that firms need to optimize their human resource competencies by fostering a positive attitude toward embracing this virtual change. For example, a review of FinTech literature shows that the banking sector has been the main focus of most research to examine the impact of technological change. The financial services industry in the US (year) stated that finance and insurance contributed 7.4% (or $ 1.5 trillion) to the United States Gross Domestic Product (GDP) in 2018. As a result, substantial economic activities and job creation resulted from the high growth of the financial sector in the United States (“The Financial Services Industry in the United States,” n. d.). In 2020, the financial sector's contribution to US GDP climbed to 22.3% (“Percentage added to the Gross Domestic Product (GDP) of the United States of America in 2020, by industry,” 2021). Since the COVID-19 lockout, customers have used finance apps 72% more frequently in Europe alone [3]. Fintech venture capital investment in the United States has maintained a high (US $ 12.9 billion), on track to surpass the 2019 record. Most of this investment (US$ 8.6 billion) came from the United States, where investors saw Fintech as a big development vector. Stripe raised US$850 million, Chime raised US$700 million, and B2B payments provider AvidXchange raised US$388 million, making payments the hottest industry for venture capital investment. Other late-stage fintech businesses raised significant fundraising rounds, including wealth-tech Robinhood (US $430 million) and cryptocurrency company (US $300 million) [4].

The FinTech industry expansion results in a shift in consumer behavior towards their involvement in using technology for ordinary payment transactions. Technology advancements have transferred consumers' traditional methods of obtaining services and commodities to online platforms. This resulted in the dismissal of business personnel unable to participate in technological change adaptation. Similar studies show that changing the environment by adapting to the updated procedures can make the banks more resilient, as the Islamic banks proved compared to conventional banking in the global crisis of 2007–2009 [5]. A study by Ref. [6] revealed the increased use of technology during COVID-19 in the US. The wavelet approach's findings make it clear that US COVID-19 cases were a significant factor in the return of bank indices at the start, middle, and end of the study period. With the rise in COVID cases, US banks reacted rapidly.

Additionally, individuals can feel uneasy about the stability of the banks, which ultimately decreases the return of bank indices. Although it has a less significant effect than US COVID-19 cases, the rise of worldwide COVID-19 cases influences US banks. Additionally, during the epidemic period, the US banking indexes are strongly and negatively impacted by both stock market volatility and the unpredictability of US economic policies.

The financial services researchers argue that they must focus on FinTech adoption. This need for change is emphasized even more by mentioning that the traditional financial sector vastly differs from FinTech in its adoption; for example, FinTech offers opportunities to enable people by lowering costs, increasing transparency, removing intermediaries, and increasing the availability of financial information [7]. As a result, it is believed that traditional financial services research cannot be applied to FinTech in the current technological era. The motivation of this study is to identify the bankers' competencies that they need to implement FinTech.

The above discussion indicates a growing amount of empirical research focusing on FinTech. However, most studies focus on new FinTech startups worldwide. Only a few academics have researched the requisite competency capacity of human resources for FinTech adoption. Banks appear to have a skills shortage, exacerbated by shocks like COVID-19 and Brexit. The demand for talent in the FinTech industry is increasing significantly. A company's talents are the individuals who have the potential and competencies to help the firm grow [8]. Moreover, despite the economic significance of the FinTech industry, the number of research focusing on required human resource competencies for adoption in banks is scarce.

This study adds to the existing literature by investigating the requisite human resource competency potential for Fintech adoption. The FinTech business's many innovation services discovered that innovative thinking and practical abilities are the true inventive talent required by this industry [9]. Companies regard their talented people as assets that contribute to strategy by keeping the organization ahead of the competition. Organizations profit from staff skill development [10]. This study examines employee competency potential as an independent variable and its impact on FinTech adoption, service innovation, and FinTech business growth. Current research focuses on new FinTech startups worldwide and their adoption, implying the need for studies assessing and identifying human resource competencies that will assist banks in addressing future Fintech requirements. Understanding abilities and talents in FinTech organizations are seen as critical in this day and age [11]. Third, while there have been numerous studies on the relationship between FinTech adoption and service innovation [12], service innovation, and FinTech firm growth [13], By extending this, this study makes a unique contribution by identifying the required competency potential with FinTech adoption, service innovation, and FinTech firm growth and as a result, taking into account the request for essential FinTech competency potential research in the United States. The current study empirically analyses the association between a firm's human resource competency potential and FinTech adoption. The influence of service innovation on FinTech business growth is also investigated to evaluate the long-term Effect of human resource competence potential on FinTech firm growth. Understanding competencies and talents in FinTech companies are essential in this current era [11]. This study will attempt to fill a gap in academia, as there are few studies in this field.

## 2 Literature review

The dynamic approaches of Human Resource Management (HRM) and its encouraging influence on employee performance to match the emerging challenges in the FinTech industry is emphasized throughout the HRM literature by numerous models and theories, for example, the Technology Adoption Model (TAM) [14], Theory of Reasoned Action (TRA) [15], Consumer Theory (CT) [16]. The current model is grounded on the Technology Adoption Model (TAM), Theory of Reasoned Action (TRA), and Consumer Theory (CT). In an organization, when employees have the required competency to use the technology, then the process of adoption becomes easy as the growing demand for FinTech professionals is increasing daily. The firms seek individuals who have education in the field of FinTech [17]. A new leadership style built on power and excellence is needed in the fintech industry. As a result, a new form of digital leadership that promotes change at the top is emerging. Work From Home (WFH) environments can be supported by their vibrant company culture and the broad range of business-enabling technologies, as well as newly virtualized business transactions and interactions. Giving organizations very effective resource management will help them in automation, for instance, expediting procedures while lowering errors.

Additionally, it reroutes human labor from low-value, repetitive jobs to higher-value ones that machines cannot complete, improving working conditions and employee and consumer experiences [18]. The rapidly developing financial technology (FinTech) industry comes with several Human Resource (HR) difficulties. Technological improvements and digital innovation have impacted the approach to creating business models and work designs, which has increased the responsibilities of Human Resource Management (HRM). An “always-on” work mode has been brought on, in particular, by the rising demand for digital innovation platforms for internal inventions in the spread of the coronavirus pandemic (COVID-19) outbreak [19]. In general, the HR department and people management will face several issues due to these new technologies. Organizations will need to develop a strategy for using these technologies to increase efficiency and decision-making while mitigating potential adverse effects on people.

**Fig. 1:**
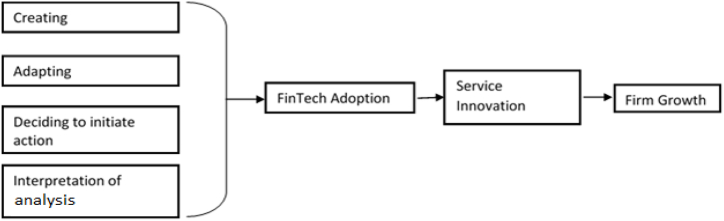
Theoretical framework.

Human resource management and technology cannot be detached. First, it was revealed that digital platforms are widely used in the workplace and are essential to online marketplaces like Amazon or eBay and labour market platforms like Uber and Freelancer.com [20]. Secondly, artificial intelligence and machine learning are widely used for data analysis, pattern recognition, and prediction in the published findings. Third, robotics was perceived to impact employment as industrial robots have increasingly taken on routine jobs that factory workers typically perform. Fourth, it has been seen that augmented and virtual reality (AR and VR) are becoming more and more important. Fifth, wearable technology is being used increasingly in the workplace to raise employee awareness of their wellness, monitor their progress, and develop strategies for maintaining engagement. The use of blockchain for transactions and information sharing that demand a high level of security is the last use case [21]. The current study investigates the effect of competency potential on service innovation while examining FinTech adoption as a mediating variable and extending its Effect on FinTech firms' growth by drawing on the Technology Adoption Model (TAM), Theory of Reasoned Action (TRA) and Consumer Theory (CT). Fig. 1 The conceptual framework is displayed, and the associated hypotheses are identified.

### 2.1 Human resource competency potential and FinTech adoption

Human Resource (HR) competencies support organizations since these competencies of HR professionals are significant to organizational performance. These competencies and skills include ideas, programs, and activities that lead to optimal organizational performance [22]. Numerous types of research on competence have used three methods to study the impact on organizational performance [23]. The behavioral technique emphasizes characteristics of past cognitive capabilities, such as self-awareness, self-control, and social skills [24,25]. The competency-based method focuses on competence as a necessary condition for task completion, restricting the competence period to perform required skills and knowledge [26,27]. A multidimensional/holistic approach to evaluating expertise refers to the individual and organizational capabilities required to achieve a goal [28].

There are several viewpoints and methods of perceiving competency. The competency model is a behavior job description that addresses the knowledge, organization-specific skills, and traits necessary to achieve adequate performance [29]. According to Ref. [2], knowledge is the sum of all competence group components (social, professional, and personal), personality, personal qualities, and abilities core skills. According to Ref. [30], four things must be evaluated when considering a candidate for the position: Empower employees to use their existing skills to reflect the current business condition. Employees must have viable career paths and development opportunities, including training for new skills. Furthermore, HR expertise, change management, and IT experience are also essential for organizational performance.

Reference [23] study revealed that grounded on your experience; you must be able to determine skills based on practice and place labels on them. In the behavioral approach (USA), personal characteristics and the application of behavioral capabilities to achieve more excellent presentations are emphasized. The function approach (UK) is more concerned with a multidimensional and holistic perspective on work competence. In contrast, the multidimensional & holistic approach (Germany, France, Austria) focuses additionally on the analytic notion of capability. Furthermore, to create a competence model for digital banking that includes legacy competencies. Reference [31] constructed a competency model for online banking that incorporates legacy skills. In the third stage, knowledge workers must apply their abilities to solve complex problems like artificial intelligence. According to a study by Ref. [2], there are numerously skilled employees for various commercial tasks in Poland. Leadership also demands developing the capacity to lead others, build vision and strategy for the future, set goals and plans, and influence how people think about things that matter to them personally and globally. Leadership skills include acquiring insight into one's values, fostering a sense of purpose in employees through leading by example, encouraging innovation from individuals working together toward the ability to work with a variety of processes, the complexity of the scope of work, and capability to collaborate/interact with machines. Employee management is critical to organizational performance and competitiveness. Reference [32] holistically screen out employee core competencies for Industry 4.0. The required technical capabilities include state-of-the-art understanding, technical skills, process comprehension, etc.

Good writing and public speaking skills are also required. In addition, there is a need for strong computer programming abilities and a thorough grasp of Information Technology (IT) safety. CEB Inc. an international technology firm that provides services to businesses all over the world, provides the SHL Universal Competency Framework (UCF) [33]. Competency models are often constructed on the foundation of knowledge. The framework has three hierarchical levels. The first is known as the “Big Eight” because it focuses on eight critical competency factors contributing to job performance. The eight competence areas were followed by 20 competency dimensions, which divided the eight groups into additional categories. The Competency Framework provides a broad view of competencies, allowing you to define specific subject-related competence models [34].

Meanwhile, Ref. [34] adapted the SHL UCF in their study for industry competence needs, and they used a combination of tests and interviews to evaluate a company's employees. The dimensions of SHL UCF that the FinTech industry professionals prioritize are creating, adapting (to change), deciding to initiate action, and interpreting analysis. FinTech adoption can only occur when the firm employees can understand and learn this new technology. Based on the earlier discussion, our first proposed hypotheses are as follows:

H_1_: Creating has a positive relationship with FinTech Adoption.
H_2_: Adapting (to change) has a positive relationship with FinTech Adoption.
H_3_: Deciding to initiate action has a positive relationship with FinTech Adoption.
H_4_: Interpretation of analysis has a positive relationship with FinTech Adoption.

### 2.2 FinTech adoption and service innovation

Industry 4.0 is impacting the utilization of technology to gain businesses and economic growth, thus creating a virtual change that requires employees' skills to handle FinTech. It is observed that the FinTech and banking industries rely immensely on the automation process, leading to the adoption of FinTech. There is a probability that FinTech is becoming one of the most extensive labor transitions in the years to come, with major changes in the skills set [35]. FinTech adoption by the consumers poses a threat to the banks as their consumers demand the same service from the bank. Unfortunately, banks cannot meet this demand because of a lack of knowledge in this field [36,37]. The technology offers user-friendly and faster services, making these FinTech companies acceptable and popular, thus posing a challenge to the banks [38]. FinTech companies are gaining the consumers' trust rapidly and breaking the relationship between the bank and their consumers, which results in a growing concern from the banks [39]. On the other side, banks see Fintech as a new opening to incorporate FinTech into their business settings [40]. For example, banks are now providing mobile banking to those consumers who still don't possess a bank account.

Furthermore, other digital banking platforms are also provided by banks. The FinTech Industry is rapidly growing, providing an opportunity for the banking sector to team up with these companies to take advantage of their innovative abilities. Banks lack the innovative capacity to provide digital platforms; FinTech companies use the banking system instead of developing a new one. When the banks and FinTech companies collaborate, it leads to emerging open banking systems [41]. Banks strategically acquire FinTech companies to avoid the cost of developing the system and adopt an intelligent approach to stay ahead of the competition [42]. These are a few factors that help in determining the adoption of innovative FinTech technology, such as internet servers, training, subscriptions of a mobile telephone, research and development, working with other companies, in particular the firm size and the technical skills of the employees [43,44]. Hence the proposed hypothesis is as follows,

H_5_: Technology adoption has a positive relationship with service innovation.

### 2.3 Service innovation and FinTech business firm growth

The banking business's role in a country's economic development and sustainability is critical. As a result, banks must provide value to shareholders by redesigning procedures and services to stay competitive and achieve excellence in the banking business. Organizations can expand by linking stakeholders and service delivery using cutting-edge technology-enabled goods and services [45]. The fourth industrial revolution has had an impact on several industries in business. One of the industries that help industrial development progress is financial services Industry 4.0. The evolution of FinTech, for example, is an essential consideration when planning for the future [46]. FinTech is expanding rapidly in numerous sectors and bringing new inventions in goods and services using cutting-edge technology [47,48]. Large-scale machines that utilize the Internet of Things (IoT) for financial efficiency are parts of FinTech [49]. The presence of FinTech may help shrink the gap between demand and supply in small businesses by providing new business models based on information technology and improving existing financial institutions' services [43].

The consumer theory explains how consumers make spending decisions based on their budgets and preferences [16]. According to this theory, the new services replace the old ones to meet the consumers' demands. FinTech services play the same role as the old services, but the only difference is that they use technology [50]. This assures the consumer's trust in technology as FinTech provides a safe transfer platform, thus building positive associations with improved financial performance. Furthermore, this trust helps the banks strengthen their financial performance by gaining more customer deposits. From the perspective of this theory, the impact of new FinTech services on the performance of the banks helps in growth. Several studies have confirmed the positive effects of service innovation on the bank's performance.

Reference [51] found a significant strong relationship between the use of mobile money on bank performance from 2009 to 2015. The research revealed the adoption of technology for making online transactions as one of the aspects of FinTech and profitability, stability, and efficiency to study the bank's performance. In addition, another study was done from 2010 to 2017, which examined the impact of innovations in technology such as mobile banking, internet banking, investments in software, and ATMs on the performance of a Lebanese bank. The study results revealed a significant positive impact of investments in Internet banking and ATMs on the bank's performance [52]. Moreover, Reference [53] claimed that FinTech could bring a phase of cost economies that was elusive to banking for a long time. Hence, we propose the following hypothesis.

H_6_: Technology adoption has a positive relationship with service innovation.

### 2.4 Mediating role of service innovation in the relationship between Fintech Adoption and FinTech Firm Growth

Financial services are transformed because of digital innovations pressuring banks to adopt FinTech. A host of new FinTech companies have sprung up which can meet the customers' demands by providing digital platforms for transactions [54]. Indeed, banks are strategically closing the gap by digitalizing their internal procedures and service innovation to meet customer demands [55,56]. The pandemic has accelerated this digital transformation, and FinTech companies seem to grow very rapidly in those financial markets with less developed financial systems [[56], [57], [58]]. Furthermore, the countries with more restrictions on mobility have experienced a large shift toward using financial applications [58].

According to Ref. [41], an open banking system is developed because of collaborations between FinTech companies and banks. Since the banks lack the human resource skills to adopt FinTech when this merger takes place, it allows banks to implement their service innovation through FinTech adoption better, thus resulting in optimal performance [59]. The COVID-19 pandemic has accelerated the use of FinTech, thus pressuring the up to 22% of insurance, asset, and wealth management companies to adopt new business models [60]. The growth of companies that use the robotic automation process for tasks related to banking is seeing a one hundred percent return on their investments [61]. According to research by Ref. [62], America FinTech's new startups were eight thousand seven hundred and seventy-five in the year 2021, followed by 4765 startups in Asia-Pacific in 2020 [63].

Furthermore, Europe, the Middle East and Africa had 7835 startups in 2020 [63]. FinTech companies are rapidly growing in the United States and Canada as FinTech's biggest segment is digital payments, valued at over $1.2 trillion in 2021 [64]. Other than this, sixty per cent of credit unions and around 49% of United States banks believe collaboration with FinTech companies is essential [65,66]. The service innovation in banks has led them to adopt FinTech, which they observed will increase their performance. There seems to be a scarcity of research in this field. Hence, based on the above discussion, the following hypothesis is proposed,

H_7_: Service Innovation mediates the relationship between Fintech Adoption and FinTech Firm Growth.

## 3 Data and methodology

### 3.1 Scale and measurement

This study investigated how human capital dimensions influence FinTech adaptation (FA). Adding more, how the service innovation (SI) plays the intervening role between the FA and FinTech firm growth (FG) in the banking sector of the USA. For this purpose, data were collected from the USA banking sector between July and September of 2021. Respondents were screened with a screening questionnaire based on employees' previous experience with FinTech adaptation. Data was collected physically and via a google form. Five Likert-adapted scales were used to quantify each construct, see Table 1. The adapted questionnaire was altered to fit the banking sector as per [67] suggestions.

**Table 1:** Scale and measurement source.

Variables.	Source	No Item.
Creating	[68]	7
Adapting (to change)	[68]	5
Decide to initiate an action	[68]	4
Interpretation of analysis	[68]	4
Fintech adoption	[69]	3
Service Innovation	[70]	5
Firm growth	[71]	6
**Total variable 07**	**Total items =**	34

### 3.2 Sampling and data collection

The study target respondents were managerial level employees. Data was gathered from middle and upper management with an online survey and intercept sampling. The KMO value was 7.17, which shows no issue with the sample size. For data collection, we approached more than 55 banks in the USA as per convenience. The questionnaire was distributed among 350 employees, and 329 responses were received. After removing outliers, 311 respondents responded and were chosen for further investigation.

### 3.3 Data analysis

The conceptual model was tested using Structural Equation Modeling (SEM). Reference [72] suggest the Smart PLS-SEM analyzes the complex structure model. Secondly, they suggest Smart PLS-SEM best investigate the whole model simultaneously. At the same time, they also recommend PLS-SEM because of its power to calculate regression and confirmatory factor analysis. Under the rationality of these three suggestions, we used Smart PLS software version 3.3.3 in this research. While the descriptive statistics of the data are presented in Table 2 below.

**Table 2:** Skewness and Kurtosis.

Items	Mean	Standard Deviation	Excess Kurtosis	Skewness	Items	Mean	Standard Deviation	Excess Kurtosis	Skewness
FG1	3.363	1.102	−0.666	−0.149	IA1	3.257	1.116	−0.721	−0.089
FG2	3.354	1.161	−0.746	−0.249	IA2	3.28	1.071	−0.891	−0.087
FG3	3.466	1.125	−0.82	−0.215	IA3	3.277	1.168	−0.811	−0.248
FG4	3.505	1.151	−0.939	−0.285	IA4	3.373	1.188	−0.741	−0.372
FG5	3.495	1.237	−0.882	−0.414	C1	3.283	1.075	−0.901	0.058
SI1	3.254	1.062	−0.731	−0.036	C2	3.35	1.08	−0.909	−0.041
SI2	3.28	1.188	−0.756	−0.208	C3	3.257	1.125	−0.909	−0.084
SI3	3.415	1.15	−0.734	−0.274	C4	3.363	1.203	−0.898	−0.261
SI4	3.389	1.173	−0.842	−0.278	C5	3.534	1.145	−0.5	−0.491
FA1	3.241	1.065	−0.808	−0.013	C6	3.559	1.16	−0.599	−0.437
FA2	3.196	1.077	−0.923	0.068	AD1	3.325	1.097	−0.739	−0.128
FA3	3.209	1.254	−0.972	−0.135	AD2	3.331	1.089	−0.933	−0.072
FA4	3.36	1.218	−0.772	−0.363	AD3	3.347	1.193	−0.896	−0.217
FA5	3.302	1.113	−0.601	−0.267	AD4	3.412	1.213	−0.885	−0.32
FA6	3.447	1.196	−0.66	−0.429	AD5	3.408	1.161	−0.618	−0.396
DTI1	3.376	1.141	−0.679	−0.319	CO1	3.572	1.07	−0.485	−0.442
DTI2	3.302	1.136	−0.923	−0.072	CO2	3.579	1.093	−0.479	−0.455
DTI3	3.267	1.292	−1.021	−0.237	CO3	3.598	1.132	−0.431	−0.525
DTI4	3.386	1.293	−0.908	−0.397	CO4	3.643	1.081	−0.043	−0.711
					CO5	3.572	1.076	−0.253	−0.546

Source: Author Calculations.

### 3.4 Study variables

The independent variables used in this study are the dimensions of human resource competency: creating, adapting (to change), deciding to initiate action, and interpreting analysis. The dependent variable is FinTech adoption, service innovation, and firm growth. Where service innovation is a mediating variable. The human resource competency variables are selected based on the ability of the human resource of banks to adapt to change. For example, to adopt financial technology, bankers should have creativity and can adjust to this changing environment. Similarly, bankers have to initiate action. That is, they should learn the technology and interpretation of the analysis selected because, without the ability to interpret research, the bankers cannot make any managerial decisions about deciding the course of action [34].

The dependent variable is FinTech adoption, as the bankers update their competency, which will help them adopt the technology. The service innovation is a mediating variable selected because the banker's competency development will help in service innovation as it is stated earlier that the ability of creativity is required. The dependent variable is firm growth, and the competency development of the human resource, along with adoption and service innovation, will generate firm growth [[69], [70], [71]].

According to Technology Adoption Model (TAM), the users accept the computer system only when they perceive it to be easy to use and valuable. This study is based on the adoption of technology by bank employees since the external environment imposes this requirement. The employees of banks have to adopt technology for the survival of their banks [14]. The theory of reasoned action is used because this study focuses on changing the pre-existing behaviours of employees toward adopting technology. Similarly, the service innovation will come from the ability created by the bank employees [15]. The consumer theory is used because it suggests how consumers decide their preferences and budget. The firms will grow when the banks can provide services that the consumer will prefer [16].

## 4 Results

### 4.1 Measurement model

**Fig. 2:**
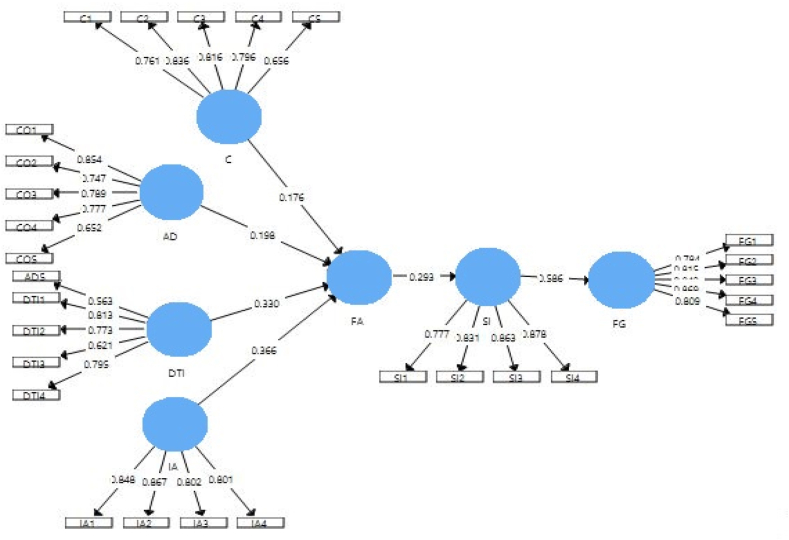
Measurement model assessment.

Statistics are used to assess the conceptual model to approve further action based on the output of the PLS-Algorithm. These outputs of statistical results focus on construct reliability, validity, collinearity, discriminant, and convergent validity. The calculation findings indicate that all measurement model results meet their minimum criteria of threshold values. For example, see Fig. 2 and Table 1. The factor loading values of each item are more significant than 0.5, Cronbach alpha values are greater than 0.706, and the AVE value is more than 0.5. These results indicated no convergent validity and reliability issues [73].

According to Ref. [74], most studies used Fornell and Larcker criterion to investigate the discriminant validity in any investigation. In this study, Table 4 shows the results of the Fornell and Larcker criteria. All diagonal values that are the square root of AVE are higher than other values of row and column or correlations values. According to Ref. [72], there will be no discriminant validity issue if AVE's square root is greater than correlation values. In Table 3, it is shown that creating has the lowest value of AVE square root of 0.711. In contrast, in correlations, FinTech Adoption and analysis interpretation have the highest value of 0.704, indicating that Fornell Larcker criteria meet [72] criterion. Assessment of measurement model is shown in Fig. 2.

**Table 3:** Reliability analysis.

Construct	Cronbach Alpha	Composite Reliability	Average Variance Extracted
AD (Adapting)	0.823	0.876	0.588
C (Creating)	0.779	0.848	0.506
DTI (Deciding to Initiate action)	0.760	0.841	0.518
FA (FinTech Adoption)	0.866	0.900	0.603
FG (FinTech Firm Growth)	0.882	0.714	0.679
IA (Interpretation of analysis)	0.849	0.898	0.688
SI (Service Innovation)	0.858	0.904	0.702

**Table 4:** Fornell Larcker criteria.

	AD	C	DTI	FA	FG	IA	SI
**AD**	0.767						
**C**	0.235	0.711					
**DTI**	0.323	0.535	0.720				
**FA**	0.460	0.597	0.688	0.777			
**FG**	0.147	0.329	0.429	0.388	0.824		
**IA**	0.314	0.541	0.545	0.704	0.326	0.830	
**SI**	0.100	0.304	0.327	0.293	0.586	0.317	0.838

Source: Author Calculations.

Note! Fig. 2 indicates the factors loading between the items and respective construct (rectangle and oval shape), while the beta value is indicated in the structure model.

### 4.2 Multicollinearity

The VIF values investigate the multicollinearity among the construct and items. The outer VIF indicates the multicollinearity among the items. See Table 5a. While the inner VIF Table 5b shows the multicollinearity among constructs. A greater VIF than 10 indicates multicollinearity [75], while Table 5a, Table 5ba and 5b indicate no VIF value greater than 10, indicating no multicollinearity problem.

**Table 5a:** Outer VIF.

Items	VIF	Items	VIF	Items	VIF
AD5	1.112	DTI1	2.108	FG1	1.777
C1	1.81	DTI2	1.968	FG2	1.973
C2	2.301	DTI3	1.301	FG3	2.415
C3	1.91	DTI4	1.797	FG4	2.655
C4	1.841	FA1	3.557	FG5	2.027
C5	1.324	FA2	2.818	IA1	2.808
CO1	2.239	FA3	2.054	IA2	2.967
CO2	1.71	FA4	3.193	IA3	1.754
CO3	1.818	FA5	2.222	IA4	1.727
CO4	1.715	FA6	1.472	SI1	1.642
CO5	1.375			SI2	1.892
				SI3	2.492
				SI4	2.611

**Table 5b:** Inner VIF.

	FA
**IA**	1.667
**DTI**	1.66
**C**	1.602
**AD**	1.151

Source: Author Calculation.

### 4.3 Structural model

**Fig. 3:**
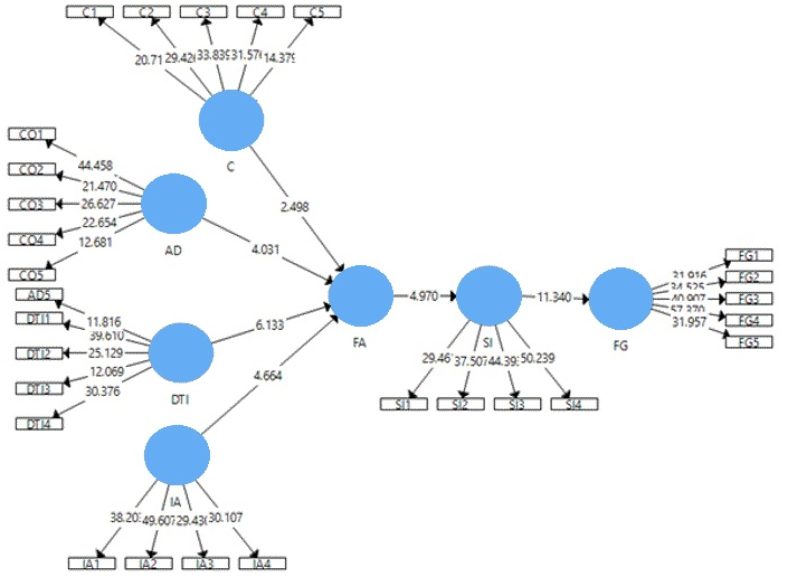
Structural model assessment.

Fig. 3 and Table 6 illustrate the association among several variables of the conceptual model [72]. explain that if the “t” value is greater than 1.96 and the significant value (‘P”) value does not exceed 0.05, it is confirmed that the specific independent variable has a significant impact on the dependent construct. The statistical study results in Table 6 proposed that all variables are significantly positively related. Besides this, under the specific indirect Effect, results show that conceptual model constructs also play the mediation relationship. For example, see Table 6 Path Model.

**Table 6:** Path model.

	Coefficient	T Statistics	P Values	Hypothesis Accepted/Rejected
Hypothesis				
AD → FA	0.193	4.031	0.000	Accepted
C → FA	0.182	2.498	0.013	Accepted
DTI → FA	0.329	6.133	0.000	Accepted
FA → SI	0.295	4.970	0.000	Accepted
IA → FA	0.365	4.664	0.000	Accepted
SI → FG	0.590	11.340	0.000	Accepted

Source: Author Calculation.

### 4.4 Blindfolding

According to Ref. [72], blindfolding indicates predictive relevance. Further, the Q^2^ greater than zero indicates that the path model has the prediction power. As shown in Table 7, all Q^2^ values are greater than zero, so this study's path model has no issue with predictive relevance.

**Table 7:** Blindfolding.

	SSO	SSE	Q^2^ (=1-SSE/SSO)
**AD**	1555.000	1555.000	
**C**	1866.000	1866.000	
**DTI**	1555.000	1555.000	
**FA**	1866.000	1118.909	0.400
**FG**	1555.000	1200.005	0.228
**IA**	1244.000	1244.000	
**SI**	1244.000	1173.187	0.057

Source: Author Calculations.

## 5 Discussion

This study has greatly influenced human resource management practices. The study's findings support the previous work of [33,34] from a theoretical standpoint and confirm the relationship between employee competencies such as creating, adapting (to change), deciding to initiate action and interpreting analysis with FinTech adoption. Further, the mediation role of service innovation on the firm growth because of employee competencies to adopt FinTech is empirically proven to be valid. These results are consistent with the findings of [33,34,38,45,46,51]. This research contributes to a better understanding of the investigated relationships in United States banks. Investigating the bank's employee competencies for successful FinTech adoption has improved the understanding of banks towards acquiring employees' competencies for service innovation, which can contribute to their growth. Future research is required in the United States banking industry.

This study's findings shed light on FinTech adoption by emphasizing the development of the right employee competencies. The intermediary linkages between acquiring the right competencies for FinTech adoption for firms' growth have not been established. The study's findings validate the relationships, particularly when employees with the right competencies are better [33,34]. This research establishes that a significant amount of effort is put into acquiring the right competencies, and a bank can take benefit from employees' long-term potential. The bank employees can take advantage of this practice when they know their competencies which are essential in FinTech adoption. These competencies contribute toward service innovation, thus increasing the firm's growth.

Furthermore, the bank employee must adapt to the changing environment as FinTech has revolutionized the financial industry. Other than this, employees' ability to decide to take initiative actions will serve as an added advantage. The bank employee's ability to create and interpret using technology is the bank's required skill. The findings of this study show that human resource competencies have a chain effect on firms' growth, with service innovation acting as a mediator between the two in the banking industry of the United States. These results align with [33,34], who demonstrated the valuable contribution of human resource competencies toward FinTech adoption, which positively influences the firm's growth. Consequently, employees quickly develop abilities to adopt FinTech and contribute toward service innovation. The findings show that FinTech adoption through identifying the right human resource competencies adds valuable knowledge for professionals in Human Resource Management (HRM).

This study indicated that employee competencies play a crucial role in FinTech adoption. The employees working in banks are not equipped with the technical knowledge, which threatens the banks as FinTech makes it easy for consumers to make quick transactions. Consumers don't have to wait for the bank's lengthy procedures, making them switch to a faster and safer medium of making their financial transactions. This study provided empirical evidence from the banking industry of the United States that banking is revolutionizing by upgrading its employee's competencies as now it is proving to be the only way for them to survive. The positive findings suggested that employees must adapt to the changes in the working environment. Along with this, they must be creative, take the initiative and have strong interpretation competency.

Moreover, this study suggests that banks have to invest in training and focus on acquiring the right competencies in their employees. FinTech is a service innovation that provides easy access to consumers' financial needs. Banks must understand this gap filled by FinTech and equip their employees with the required skills to serve their current markets. This study indicates that the employees who enjoy new experiences and are open most to new approaches and work methods will adapt faster. Furthermore, employees who can probe information for potential errors in the analysis will be more beneficial. Depending on the situation, employees who enjoy variety and change and consider new and established methods can quickly adopt FinTech. Banks must focus on acquiring the competency of interpretation and analysis as the employees must understand the latest technology, so they need these competencies. Few studies in this field have focused on employee competencies for FinTech adoption. Furthermore, rather than looking at FinTech adoption as a single entity, the banks must focus on developing and acquiring the right employee competencies.

## 6 Conclusion

This research bridges the gap by demonstrating that successful development and acquisition of human resource competencies can support the banks in FinTech adoption. The banking employees are not equipped with technical knowledge. Because of this lack of knowledge, they cannot serve their consumers as their consumers are satisfied with using FinTech. The banks of the United States have to understand this gap, and their right human resource competencies can fill it. The bank employees must adapt to the new technology by upgrading their knowledge.

Furthermore, employees can use their creative skills in service innovation [45]. The employees must go the extra mile by taking the initiative and using their interpretation competencies to assess the environment to create a workable solution for their consumers [33,34]. Our research shows that the need to upgrade bank employees' skills is now much crucial for the survival of the banking industry as FinTech can remove banking operations altogether. Strong regulatory measures and alternate survival strategies are thus needed for US banks to sustain their stability. The US government may enact laws that will help US banks survive.

This study's managerial implications confirm that the banks must upgrade their employee's competencies by conducting training [29]. Furthermore, the banks can hire FinTech employees who already possess the technical skills and can better handle the issues faced by the banking sector. Reference [9] suggest that employee creativity is crucial to service innovation. The banking sector is suffering because of a lack of competencies in technology. The managers must understand the demands of FinTech and equip their employees with the right competencies to face the challenges of FinTech adoption. This study, like other studies, has limitations. Since this is a cross-sectional study, it cannot address the causality issue. Secondly, this research is limited to the banking industry of the United States. This study is limited to using the Technology Adoption Model (TAM), Theory of Reasoned Action (TRA) and Consumer Theory (CT). However, maybe other theories support investigating the same subject problem. Future researchers may explore the adoption of FinTech with the help of different approaches.

This study is quantitative, and future researchers can explore the phenomena through a qualitative approach. For this purpose, they should use an inductive approach. Third, this research is focused on human resource competencies and does not consider personality traits. This research has opened up different opportunities for using the same model in other industries to generalize the results better. This competency compatibility of FinTech for bank employees is also beneficial for the consumers because as they use FinTech for their daily transactions, they need customer support which can only be provided when the employees are trained. The consumer of FinTech is tech savvy and is well aware of the technology used since it helps them save time. The only deadlock that the consumers face is the lack of awareness of bank employees. So, the social implication of bank employees developing competency compatibility with FinTech will help them serve better to their users [76].

## Author contribution statement

Conceived and designed the experiments: Sana Arz Bhutto, Yasir Jamal.

Performed the experiments: Sana Arz Bhutto, Yasir Jamal.

Analyzed and interpreted the data: Yasir Jamal, Saif Ullah.

Contributed reagents, materials, analysis tools or data: Sana Arz Bhutto, Yasir Jamal, Saif Ullah.

Wrote the paper: Sana Arz Bhutto, Yasir Jamal, Saif Ullah.

## Funding statement

This research did not receive any specific grant from funding agencies in the public, commercial, or not-for-profit sectors.

## Data availability statement

Data included in article/supp. material/referenced in article.

## Declaration of interest's statement

The authors declare no competing interests.

## Figures and Tables

**Table:** 

Constructs	Itmes	Measures	Scources
C	C1	I give some thought to potential limitations when reviewing new Information?	[68]
	C2	I consider new and established approaches when learning new tasks?	
	C3	I enjoys variety and change?	
	C4	I consider both new and established methods, depending on the situation?	
	C5	I very likely to focus on the broader picture rather than the detail?	
	C6	I consider new and established methods when establishing a vision?	
AD	AD1	I adapt to changing circumstances	[68]
	AD2	I tolerate ambiguity	
	AD3	I accept new ideas and change initiatives	
	AD4	I adapt interpersonal style to suit different people or situations	
	AD5	I show an interest in new experiences	
DTI	DTI1	I decides upon a course of action very quickly	[68]
	DTI2	I holds strong views and is comfortable with acting independently	
	DTI3	I places a high emphasis on achieving difficult targets	
	DTI4	I feel comfortable as to take charge of situations when required	
IA	IA1	I moderately likely to critically evaluate the content of written information.	[68]
	IA2	I am inclined as most others to understand the needs of an audience.	
	IA3	I may critically evaluate technical information in some situations.	
	IA4	I will probe information for potential errors in analysis.	
FA	FA1	I will continue using fintech services	[69,70]
	FA2	I have not used but would like to use fintech services soon	
	FA3	I will recommend fintech services to my friends	
SI	SI1	My bank has creative service packages.	[70]
	SI2	My bank has flexible service package options.	
	SI3	My bank is noticeably different in concept, compared to preceding services	
	SI4	My bank is totally different in experience compared to preceding services	
	SI5	My bank service is noticeably different in concept and design, compared to competing services	
FG	FG1	Our firm has experienced steady increase in sale	[71]
	FG2	Our firm market share has expanded	
	FG3	Our firm has increased the number of its employees	
	FG4	Our firm size has grown	
	FG5	Our firm assets are increasing steadily	
	FG6	There is increased output of services by our firm	
