# Supplementary material for: FinTech adoption, HR competency potential, service innovation and firm growth in banking sector
Source: Heliyon. 2023 Feb 23;9(3):e13967. doi: 10.1016/j.heliyon.2023.e13967 (PMC10006525; doi:10.1016/j.heliyon.2023.e13967)
Supplement: Multimedia component 1 [file mmc1.docx]

***Table 1***

*Scale and measurement source*

| **Variables.** | **Source** | **No Item.** |  |
| --- | --- | --- | --- |
| Creating | Hollander, P. (2008) | 7 | |
| Adapting | Hollander, P. (2008) | 5 | |
| Decide to initiate an action | Hollander, P. (2008) | 4 | |
| Interpretation of analysis | Hollander, P. (2008) | 4 | |
| Fintech adoption | (Nathan, Setiawan and Quynh, 2022). | 3 | |
| Service Innovation | (Mahmoud, Hinson, and Anim, 2017). | 5 | |
| Firm growth | (Blose, 2018). | 6 | |
| **Total variable 07** | **Total items =** | 34 |  |

**Creating**

C1 I give some thought to potential limitations when reviewing new Information?

C2 I consider new and established approaches when learning new tasks?

C3 I enjoys variety and change?

C4 I consider both new and established methods, depending on the situation?

C5 I very likely to focus on the broader picture rather than the detail?

C6 I likely as peers to take a longer-term view when developing strategy?

C7 I consider new and established methods when establishing a vision?

**Adapting**

AD1 I adapt to changing circumstances

AD2 I tolerate ambiguity

AD3 I accept new ideas and change initiatives

AD4 I adapt interpersonal style to suit different people or situations

AD5 I show an interest in new experiences.

**Decide to initiate an action**

DTI1 I decides upon a course of action very quickly

DIT2 I holds strong views and is comfortable with acting independently.

DIT3 I places a high emphasis on achieving difficult targets.

DIT4 I feel comfortable as to take charge of situations when required

**Interpretation of analysis**

IA1 I moderately likely to critically evaluate the content of written information.

IA2 I am inclined as most others to understand the needs of an audience.

IA3 I may critically evaluate technical information in some situations.

IA4 I will probe information for potential errors in analysis.

**Fintech adoption**

FA1 I will continue using fintech services

FA2 I have not used but would like to use fintech services soon

FA3 I will recommend fintech services to my friends

**Service Innovation**

SI1 My bank has creative service packages.

SI 2 My bank has flexible service package options.

SI3 My bank is noticeably different in concept, compared to preceding services

SI4 My bank is totally different in experience compared to preceding services

SI5 My bank service is noticeably different in concept and design, compared to competing services

**Firm growth**

FG1 Our firm has experienced steady increase in sale

FG2 Our firm market share has expanded

FG3 Our firm has increased the number of its employees

FG4 Our firm size has grown

FG5 Our firm assets are increasing steadily

FG6 There is increased output of services by our firm

Blose, S. (2018). *Factors hindering the growth of SMMEs in Msunduzi Municipality, KwaZulu-Natal Province* (Doctoral dissertation).

Mahmoud, M. A., Hinson, R. E., & Anim, P. A. (2017). Service innovation and customer satisfaction: the role of customer value creation. *European Journal of Innovation Management*.

Nathan, R. J., Setiawan, B., & Quynh, M. N. (2022). Fintech and financial health in Vietnam during the COVID-19 pandemic: In-depth descriptive analysis. *Journal of Risk and Financial Management*, *15*(3), 125.

Hollander, P. (2008). *Universal Competency Report*. SHL Group Limited
